# Up-regulation of liver *Pcsk9* gene expression as a possible cause of hypercholesterolemia in experimental chronic renal failure

**DOI:** 10.1007/s11010-015-2590-0

**Published:** 2015-10-19

**Authors:** Elzbieta Sucajtys-Szulc, Marek Szolkiewicz, Julian Swierczynski, Boleslaw Rutkowski

**Affiliations:** Department of Nephrology, Transplantology and Internal Medicine, Medical University of Gdansk, ul. Debinki 7, 80-211 Gdańsk, Poland; Department of Biochemistry, Medical University of Gdansk, ul. Debinki 1, 80-211 Gdańsk, Poland

**Keywords:** PCSK9, SREBF-2, LDL, LDL-R, Hypercholesterolemia, CRF

## Abstract

Dyslipidemia commonly present in patients with chronic kidney disease (CKD) has been recently linked to increased proprotein convertase subtilisin/kexin type 9 (PCSK9) serum concentration. We tested a hypothesis that increased liver PCSK9 biosynthesis could be partially responsible for the elevated circulating PCSK9 level, and subsequently contribute to hypercholesterolemia observed in subjects with CKD. Rat model of chronic renal failure (CRF) was used in the study. Animals underwent a 5/6 nephrectomy or a sham operation. Liver expression of *Pcsk9,* sterol regulatory element-binding transcription factor 2 (*Srebf*-*2),* and *β*-*actin* were quantified by real-time RT-PCR. Liver protein levels of PCSK9, LDL-receptor (LDL-R), and SREBF-2 were analyzed using Western blotting. Serum PCSK9 concentration was estimated by immunoassay. Rats with an experimental CRF as compared to pair-fed and control ones were characterized by: (a) an up-regulation of liver *Pcsk9* and *Srebf*-*2* genes expression with parallel increase of serum PCSK9 concentration; (b) a decrease in liver LDL-R protein level, and (c) an increase of serum total and LDL-cholesterol concentrations. We also found significant correlations between serum creatinine and liver PCSK9 mRNA levels (*r* = 0.88, *p* < 0.001) and between serum creatinine and circulating PCSK9 levels (*r* = 0.73, *p* < 0.001). The results suggest that a rat model of CRF is associated with an increased liver *Pcsk9* gene expression. The coordinated up-regulation of *Pcsk9* and *Srebf*-*2* genes expression suggests that SREBF-2 may play a key role in regulation of *Pcsk9* gene expression, circulating PCSK9 level, and hypercholesterolemia in experimental CRF.

## Introduction

Dyslipidemia, one of the most potent cardiovascular risk factor in patients with chronic kidney disease (CKD), significantly contributes to increased morbidity and mortality in this group of patients as compared to general population [1]. Moreover, lipid abnormalities themselves accelerate progression of CKD [2]. Therefore, an evaluation of the molecular mechanism leading to CKD-related lipid disturbances is necessary for a better understanding and treatment of the disease. In our prior studies, we have shown that hypercholesterolemia observed in experimental CRF is partially caused by the enhanced cholesterol biosynthesis associated with the up-regulation of *Srebf*-*2* and *Hmg*-*CoA* reductase genes expression as well as by a decrease in LDL-receptor (LDL-R) mRNA level [3–5]. However, in our in vivo experimental model, cholesterol biosynthesis rate was increased only twofold, while circulating cholesterol concentration was 3–4-times higher in CRF rats when compared to control [5]. Thus, one can assume that the increased cholesterol biosynthesis is not the only process contributing to hypercholesterolemia in experimental CRF rats.

Several papers published in last decade suggest that proprotein convertase subtilisin/kexin type 9 (commonly known as PCSK9; originally named NARC-1-neural apoptosis-regulated convertase-1) plays a crucial role in LDL-cholesterol (LDL-Ch) metabolism [6–9]. PCSK9 is synthesized mainly in hepatocytes (and in lesser amounts in intestine and other organs) [10] as a 74 kDa soluble zymogen (proPCSK9) that undergoes autocatalytic cleavage into propeptide (14 kDa) and PCSK9 (60 kDa) in the endoplasmic reticulum [10]. Cleavage is necessary to activate the convertase and to allow its secretion into circulation [10]. PCSK9 secreted into the bloodstream plays a crucial role in the regulation of circulating LDL-Ch concentration by binding to hepatocytes LDL-R, promoting their intracellular degradation, and preventing LDL-R recycling to the cell surface [7, 9, 11, 12]. In liver, *Pcsk9* gene expression is up-regulated by SREBF-2 and down-regulated by cholesterol [11, 12]. PCSK9 null mice display increased LDL-R level and low circulating cholesterol concentration, whereas mice overexpressing PCSK9 exhibit high circulating cholesterol concentration [13, 14]. In humans, circulating PCSK9 level correlated positively with serum total and LDL-Ch concentration [15] and there are reports suggesting that elevated serum concentration of PCSK9 may play a role in early pathogenesis of atherosclerosis [16]. Thus, measuring circulating PCSK9 level and treating patients with human monoclonal PCSK9 antibodies [17] may have clinical significance. Recently, it has been shown that patients with nephrotic syndrome or patients with advanced renal failure treated by peritoneal dialysis displayed elevated plasma PCSK9 level [18]. Furthermore, in rats with experimental nephrotic syndrome, an up-regulation of liver PCSK9 was observed [19]. We also have recently reported that circulating PCSK9 concentration is significantly elevated in CKD patients [20] and we have suggested that PCSK9 overproduction could partially contribute to its elevated serum concentration found in patients with this disease [20]. We based this assumption on our previously reported data indicating that in rat model of CRF a liver *Srebf*-*2* gene is overexpressed [5] and that *Pcsk9* gene expression is up-regulated by SREBF-2 [21, 22]. The aim of the current study was to test the hypothesis that in rats with experimental CRF, liver *Pcsk9* gene expression is up-regulated and that SREBP-2 could be involved in this process. Our findings, presented here for the first time, provide new information about coordinated up-regulation of liver *Pcsk9* and *Srebf*-*2* genes expression leading to hypercholesterolemia observed in experimental CRF.

## Materials and methods

### Animals

Male Wistar rats (10 weeks old, weight ~250 g at the beginning of the study) were used in all experiments. There were 10 animals per each studied group (CRF, pair-fed, control). Animals were kept in individual cages with free access to tap water, and with controlled lighting schedule (illuminated from 7 a.m to 7 p.m.). Experimental CRF was induced by two-stage (5/6) subtotal nephrectomy as described previously [23]. Average daily food intake was measured by the difference in weight between the amount of food provided and the amount remaining over a 1-day period. Pair-fed (sham-operated) rats received the amount of food (its composition has been described previously [24]) corresponding to what had been consumed by the matched CRF animals.

The non-fasted overnight rats were terminated (between 8 a.m. to 10 a.m.) 6 weeks after induction of renal failure. Liver was collected, and immediately frozen in liquid nitrogen for subsequent analyses of genes expression. The tissue was stored at −80 °C until the analysis. Blood samples were collected from abdominal aorta, centrifuged, and the serum was used for determination of total protein, albumin, urea (BUN), creatinine, total cholesterol (TCh), and LDL-cholesterol (LDL-Ch). All animal procedures were conducted in agreement with our institutional guidelines for the care and use of laboratory animals.

### RNA isolation

Total cellular RNA was extracted from frozen tissue by a guanidinium isothiocyanate-phenol/chloroform method [25]. The RNA concentration was determined from the absorbance at 260 nm and all samples had 260/280 nm absorbance ratio of about 2.0.

### cDNA synthesis

First strand cDNA was synthesized from 2 µg of total RNA (RevertAid™ First Strand cDNA Synthesis Kit—Fermentas UAB, Lithuania). Prior to amplification of cDNA, each RNA sample was treated with RNase-free DNase I (Fermentas UAB, Lithuania) at 37 °C for 30 min.

### Determination of mRNA levels by real-time RT-PCR

PCSK9, SREBF-2, and β-actin mRNA levels were quantified by real-time RT-PCR using Chromo4 Real-Time Detection System (Bio-Rad Laboratories, Inc; USA). Primers were designed with Sequence Analysis software package (Informagen, Newington, USA) from gene sequence obtained from Ensembl Genome Browser. The sequences of primer pairs (sense and antisense) used in this study are presented in Table 1. Real-time PCR amplification was performed in a 20 μl volume using iQ SYBR Green Supermix (Bio-Rad Laboratories, Hercules, CA). Each reaction contained cDNA and 0.3 μM of each primer. Control reactions, with omission of the RT step or with no template cDNA added, were performed with each assay. All samples were run in triplicate. To compensate for variations in the amount of added RNA, and in the efficiency of the reverse transcription, β-actin mRNA was quantified in corresponding samples as a control and the results were normalized to these values. Relative quantities of transcripts were calculated using the $2^{{ - \Delta \Delta C_{\text{T}} }}$ formula [26]. The results are expressed in arbitrary units (a. u.), with one unit being the mean mRNA level determined in the control group. Amplification of specific transcripts was further confirmed by obtaining the melting curve profiles and subjecting the amplification products to agarose gel electrophoresis.

**Table 1:** Oligonucleotide primers sequences used in this study

Gene	Primer sequence	Accession Nr
*Pcsk9*	F: 5′-TGGCTGCATGACATTGCTTCTC-3′ R: 5′-GCACTGGAGAACCACACAGG-3′	199253
*Srebf*-*2*	F: 5′-ACTGTCACTGGAGTCAGGTT-3′ R: 5′-GACCAACAGCTTCACGAAGA-3′	001033694
*β*-*actin*	F: 5`-TGTCACCAACTGGGACGATA-3 R: 5`-GGGGTGTTGAAGGTCTCAAA-3′	031144.3

### Serum PCSK9 concentration

Serum PCSK9 concentration was estimated by immunoassay method using Rat Protein Convertase subtilisin/kexin type 9 (PCSK9) ELISA kit CSB-EL017647RA from CUSABIO BIOTECH CO.

### Western blot analysis of PCSK9, LDL-R, SREBP-2

Frozen liver samples were homogenized in buffer containing 10 mM Tris–HCl (pH 6.8), 2 % SDS, 10 mM DTT, proteinase inhibitors (Sigma), and then centrifuged at 15,000×*g* for 20 min at 20 °C. Supernatants were collected, and the protein concentration was determined by Bradford assay. Supernatants, containing 20 μg of total protein, were separated by 10 % SDS-PAGE and electroblotted onto Immobilon^®^ Transfer Membrane (Millipore). The following antibodies were used: mouse/rat Proprotein Convertase 9 polyclonal antibody (AF3985, R@D SYSTEMS), polyclonal antibody against LDL-R (sc-11826, Santa Cruz Biotechnology), polyclonal antibody against SREBP-2 (sc-5603, Santa Cruz Biotechnology), and polyclonal rabbit antibody against Actin (A5060, Sigma). HRP-conjugated secondary antibody (sc-2030 and sc-2004) was obtained from Santa Cruz Biotechnology and HAF019 from R@D SYSTEMS. Immunodetection was accomplished with enhanced chemiluminescence by using Western Blotting Luminol Reagent (sc-2048, Santa Cruz Biotechnology). Band intensities of analysed proteins were quantified and normalized to the intensity of actin band, with CON value assumed as 1. Quantification of Western blots were performed using Quantity One software program (Bio-Rad Laboratories, Hercules, CA). The results are expressed in arbitrary units (a. u.), with one unit being the mean of corresponding protein (LDL-R and SREBP-2) level determined in the control group.

### Statistical analysis

The statistical significance of differences between groups was assessed by one-way analysis of variance (ANOVA) followed by Student–Newman–Keuls and by one-way analysis of variance (ANOVA) followed by Tukey’s post hoc test. The Sigma Stat software (Sigma Stat Inc.) was used. Differences between the groups were considered significant when *p* < 0.05. All data are presented as means of values ± standard deviation (SD).

## Results

**Fig. 1:**
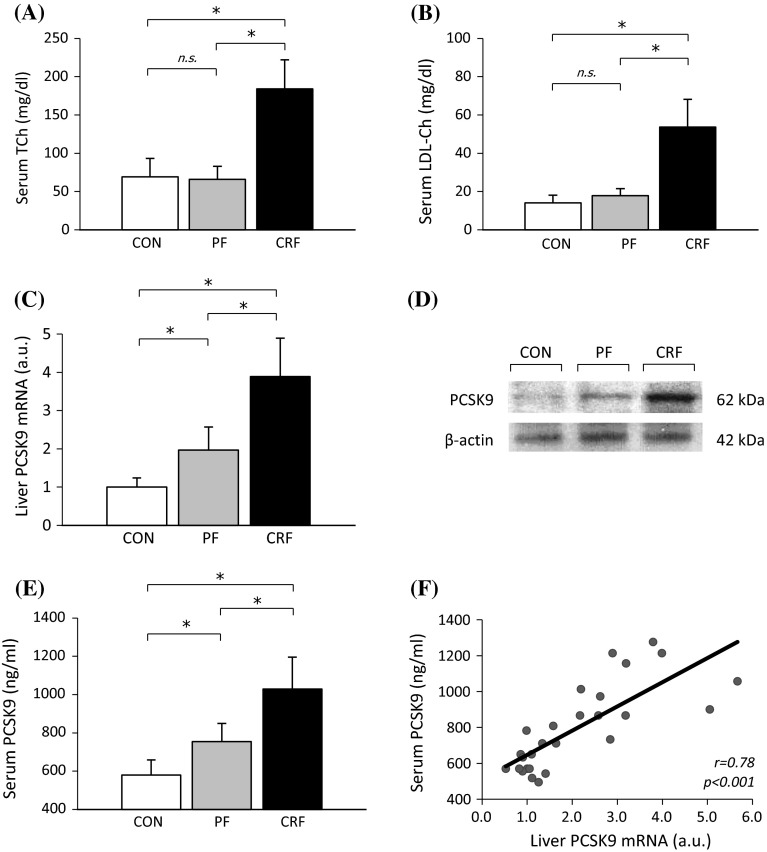
Liver *Pcsk9* gene expression and serum cholesterol and PCSK9 concentrations in control (CON), pair-fed (PF) and chronic renal failure (CRF) rats: **a** Serum total cholesterol (TCh) concentration (mean ± SD; *n* = 10 per group); **b** Serum LDL-cholesterol (LDL-Ch) concentration (mean ± SD; *n* = 10 per group); **c** Relative liver PCSK9 mRNA level expressed as arbitrary unit (a. u.) The mean ± SD was obtained from 10 rats per group; **d** The representative Western blot analysis of liver PCSK9 protein level. Actin was used as a standard; **e** Serum PCSK9 concentration determined by ELISA (mean ± SD; *n* = 10 per group); **f** The relationship between serum PCSK9 concentration and liver PCSK9 mRNA level (based on data presented in **a** and **c**). Statistics: *p < 0.001

Serum concentration of creatinine increased approximately fourfold in CRF rats as compared to control (2.4 ± 0.7 vs. 0.5 ± 0.1 mg/dl; *p* < 0.001) or pair-fed (2.4 ± 0.7 vs. 0.6 ± 0.1 mg/dl; *p* < 0.001) animals, while in control (CON) and pair-fed (PF) ones they were essentially similar (not shown). Similarly, serum concentration of nitrogen urea increased approximately threefold in CRF rats as compared to CON (47.7 ± 7.7 vs. 15.1 ± 1.2 mg/dl; *p* < 0.001) or PF (47.7 ± 7.7 vs. 14.9 ± 0.8 mg/dl; *p* < 0.001) ones (not shown). Simultaneously, serum concentrations of TCh and LDL-Ch in CRF rats were approximately threefold higher than in CON and PF ones (Fig. 1a, b). Presented data indicate that CRF induced by 5/6 nephrectomy corresponded to stage 5 CKD in patients. Positive correlations between serum creatinine and TCh concentrations (*r* = 0.82; *p* < 0.001) and between serum creatinine and LDL-Ch concentrations (*r* = 0.84; *p* < 0.001) were observed (not shown). This suggests that serum TCh and LDL-Ch levels are tightly related to progression of kidney disease.

**Fig. 2:**
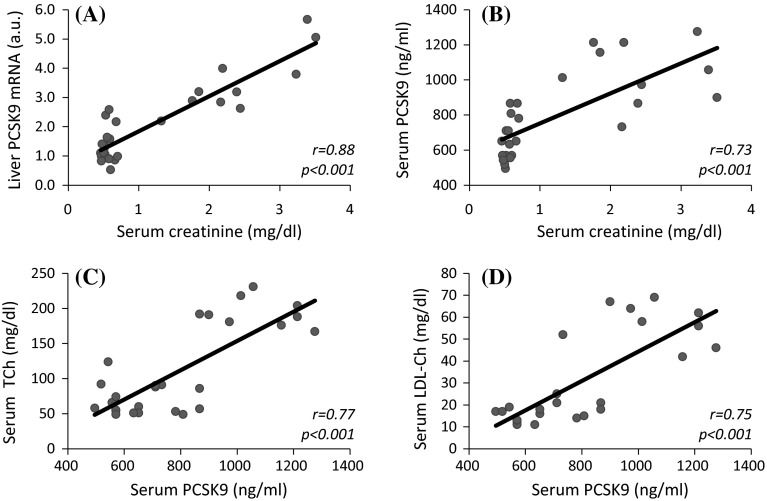
The relationships between: **a** serum creatinine concentration and liver PCSK9 mRNA levels expressed as arbitrary unit (a. u.); **b** serum creatinine concentration and circulating PCSK9 levels (determined by ELISA); **c** serum concentrations of PCSK9 (determined by ELISA) and serum total cholesterol (TCh) concentrations); **d** serum concentrations of PCSK9 (determined by ELISA) and LDL-cholesterol (LDL-Ch) concentrations

Figure 1c shows the relative PCSK9 mRNA levels in liver of CON, PF, and CRF rats. The greatest level of liver PCSK9 mRNA was found in CRF rats (approximately 3.5-fold greater that in CON and approximately twofold greater as compared to PF). The differences in liver PCSK9 mRNA levels in CON, PF, and CRF rats were paralleled by differences in liver PCSK9 protein amounts determined by Western blot analysis (Fig. 1d) and serum PCSK9 concentrations determined by ELISA (Fig. 1e). Moreover, we found positive correlations between serum PCSK9 concentration and liver PCSK9 both mRNA level (Fig. 1f) and PCSK9 protein amount (*r* = 0.93; *p* < 0.001) (data not shown). Furthermore, we observed positive correlations between serum creatinine concentration and liver PCSK9 mRNA (Fig. 2a) and circulating PCSK9 (Fig. 2b) levels. This suggests that *Pcsk9* gene expression and serum PCSK9 concentration are closely related to renal failure.

**Fig. 3:**
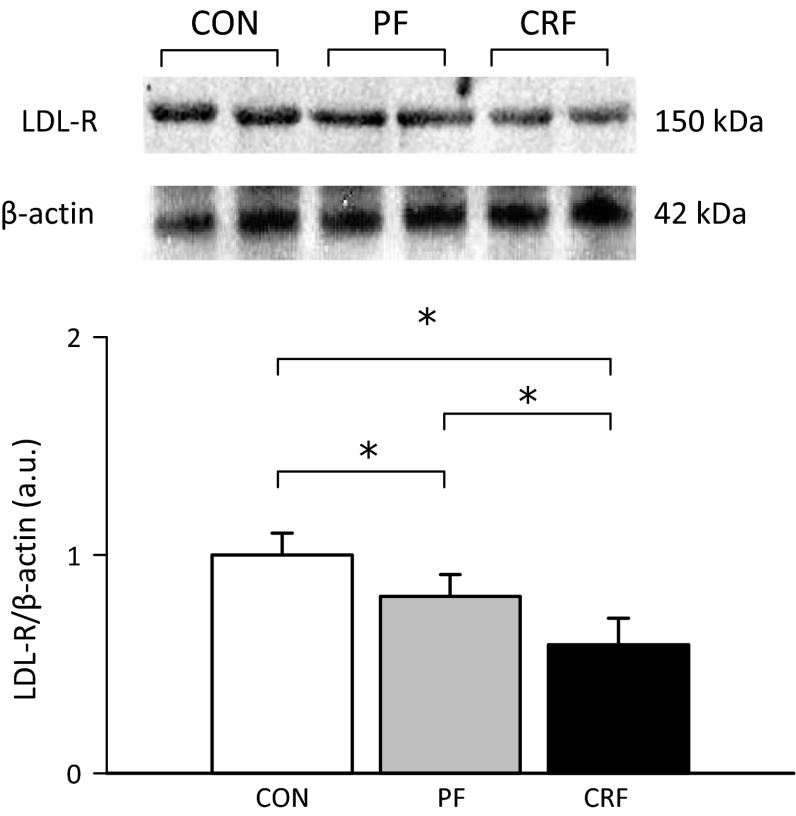
The representative Western blot analysis (*upper panel*) and group data (*lower panel*; the mean ± SD was obtained from 10 rats per group) depicting the liver LDL-receptor (LDL-R) protein level in control (CON) pair-fed (PF) and chronic renal failure (CRF) rats. Actin was used as a standard. Statistics: *p < 0.001

Serum TCh and LDL-Ch concentrations are positively correlated with a circulating PCSK9 level (Fig. 2c, d). Moreover, the increased liver *Pcsk9* gene expression and subsequently the increased circulating PCSK9 level were accompanied by a decreased liver LDL-R protein amount in CRF rats (Fig. 3). Inverse associations between a liver LDL-R protein amount and serum TCh (*r* = −0.75; *p* < 0.001) or LDL-Ch (*r* = −0.81; *p* < 0.001) concentrations were also found (data not shown). Presumably, this partially contributes to hypercholesterolemia observed in rat model of CRF.

**Fig. 4:**
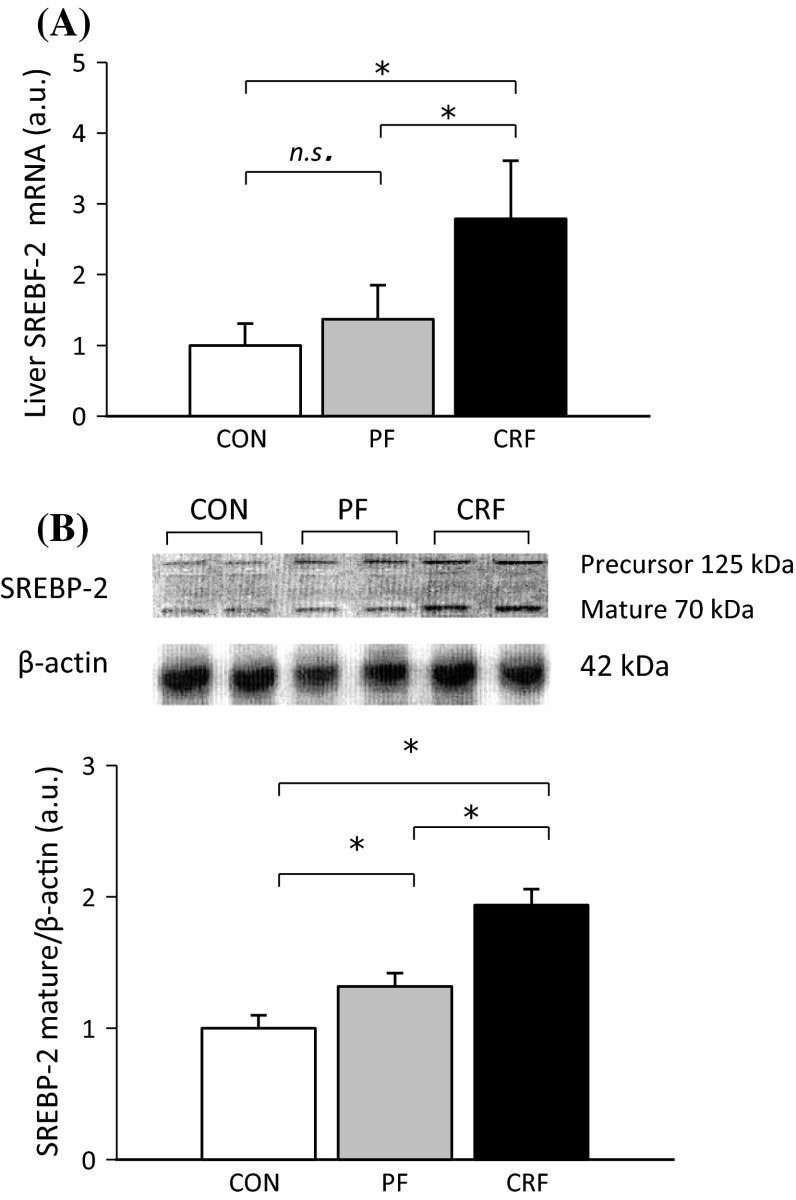
Liver *Srebf*-*2* gene expression in control (CON), pair-fed (PF) and chronic renal failure (CRF) rats: **a** Relative liver SREBF-2 mRNA level presented as arbitrary unit (a. u.; mean ± SD; *n* = 10 per group). **b** The representative Western blot analysis (*upper panel*) and group data (*lower panel*; the mean ± SD was obtained from 10 rats per group) depicting the liver precursor and mature SREBB-2 protein level. Actin was used as a standard. Statistics: *p < 0.001

**Fig. 5:**
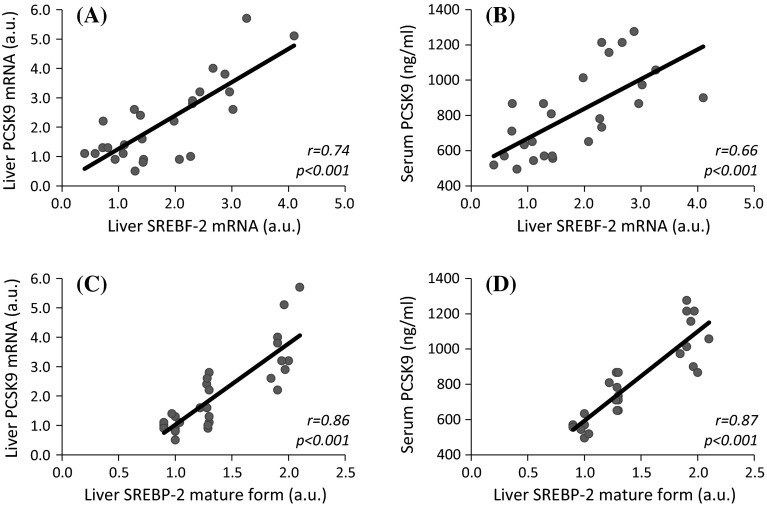
The relationships between: **a** liver SREBF-2 mRNA and liver PCSK9 mRNA levels expressed in arbitrary unit (a. u.); **b** liver SREBF-2 mRNA level expressed in arbitrary unit (a. u.) and serum PCSK9 concentration (determined by ELISA); **c** liver SREBP-2 mature form level expressed in arbitrary unit (a. u.) and liver PCSK9 mRNA level expressed in arbitrary unit (a. u.); **d** liver SREBP-2 mature form level expressed in arbitrary unit (a. u.) and serum PCSK9 concentration determined by ELISA

Since SREBP-2 (product of *Srebf*-*2*) is an important regulator of *Pcsk9* gene expression [21, 22], we determined liver *Srebf*-*2* gene expression and its association with both liver PCSK9 mRNA and serum PCSK9 levels in CON, PF, and CRF rats. Experimental CRF was associated with a significant increase of liver SREBF-2 mRNA level as compared to CON and PF (Fig. 4a). The differences in liver SREBF-2 mRNA levels were paralleled by differences in SREBP-2 protein amounts determined by Western blot analysis (Fig. 4b). The pattern of changes in liver SREBF-2 mRNA levels of CON, PF, and CRF rats resembles the changes in liver PCSK9 mRNA and protein levels (Fig. 1c, d). Finally, strong positive correlations between liver SREBF-2 mRNA and protein mature form levels versus liver PCSK9 mRNA and serum PCSK9 concentrations were also observed (Fig. 5a–d).

## Discussion

It is generally accepted that PCSK9 is primarily synthesized in liver [27] and that its circulating form is mainly cleared by the liver through LDL-R [6, 7, 9]. It means that a liver is the main organ regulating serum PCSK9 concentration. Recently, we postulated that PCSK9 overproduction could partially contribute to its elevated serum concentration found in patients with CKD [20]. In the present study, to gain a better insight into the role of PCSK9 in a development of CRF-related hypercholesterolemia, we examined liver *Pcsk9* gene expression in rat model of this disease. Moreover, we explored a possible link between liver *Pcsk9*, *Srebf*-*2* (SREBP-2 as a positive regulator of *Pcsk9* gene expression) genes expression and hypercholesterolemia observed in rats with CRF. The data presented herein prove for the first time that an experimental CRF is associated with a coordinated up-regulation of both liver *Pcsk9* and liver *Srebf*-*2.* Thus, it is likely that up-regulation of *Srebf*-*2* gene causes an elevation of circulating PCSK9 level. The increase of all liver PCSK9 mRNA and protein levels suggests that in experimental CRF, circulating PCSK9 concentration is regulated at the gene transcription level, similarly as in other pathophysiological conditions [21, 22]. As expected, the increased circulating PCSK9 concentration is associated with a lower level of liver LDL-R. The data suggest that the loss of LDL-R impairs LDL-Ch clearance, and consequently leads to serum TCh and LDL-Ch accumulation. Our data also confirm that CRF is associated with an up-regulation of liver *Srebf*-*2* gene expression.

It is obvious that we cannot exclude other mechanisms contributing to PCSK9 up-regulation found in our experimental model of CRF. As it was recently presented, up-regulation of PCSK9 gene expression could also be mediated by protein urinary loss in subjects with nephrotic syndrome [19] or by protein peritoneal loss commonly observed in peritoneal dialysis patients [18]. Data from our studies indicate lower plasma total protein (59.2 ± 7.1 vs. 66.4 ± 11.1 g/l; *p* < 0.01) and albumin (3.4 ± 0.2 vs. 4.0 ± 0.4 g/dl; *p* < 0.001) (not shown) levels in CRF rats as compared with CON ones. This is presumably due to rat model of CRF-related proteinuria, which could mediate elevation of serum PCSK9 level [28]. However, the strong positive correlation found between liver PCSK9 mRNA level and serum creatinine concentration (Fig. 2a) suggests that CRF also partially contributes to PCSK9 up-regulation. This assumption is also supported by other results, i.e., positive correlation between serum creatinine and PCSK9 concentrations (Fig. 2b) and positive correlation between liver SREBP-2 mRNA and PCSK9 mRNA levels (Fig. 5a).

All the presented data allow us to recognize that PCSK9 plays an important role in experimental CRF-related lipid metabolism disorders. Due to obvious limitations, most of these studies cannot be performed in patients with this disease. However, it should be at least considered how these findings are related to human CKD. Based on the data reported recently [20], we can only hypothesize that a similar up-regulation of liver hepatic transcriptional factor and subsequent stimulation of *Pcsk9* gene expression could be found in CKD patients.

In conclusion, the results presented in this paper indicate that experimental model of CRF is associated with an increased liver *Pcsk9* gene expression, an enhanced circulating PCSK9 concentration and in consequence, a decreased liver LDL-R level. All these contribute to CRF-related hypercholesterolemia. Coordinated up-regulation of *Srebf*-*2* and *Pcsk9* genes expression suggests that SREBP-2 may play a crucial role in regulation of *Pcsk9* gene expression and consequently contribute to hypercholesterolemia observed in subjects with CKD.
